# New directions in neurosteroid therapeutics in neuropsychiatry

**DOI:** 10.1016/j.neubiorev.2025.106119

**Published:** 2025-03-22

**Authors:** Charles F. Zorumski, Douglas F. Covey, Yukitoshi Izumi, Alex S. Evers, Jamie L. Maguire, Steven J. Mennerick

**Affiliations:** a Departments of Psychiatry, Washington University School of Medicine, St. Louis, MO, USA; b Developmental Biology, Washington University School of Medicine, St. Louis, MO, USA; c Anesthesiology, Washington University School of Medicine, St. Louis, MO, USA; d Taylor Family Institute for Innovative Psychiatric Research, Washington University School of Medicine, St. Louis, MO, USA; e Department of Neuroscience, Tufts University School of Medicine, Boston, MA, USA

**Keywords:** Allopregnanolone, Pregnenolone sulfate, GABA receptors, NMDA receptors, Major depression, Neuroinflammation, Brain networks

## Abstract

In recent years three neuroactive steroids (NAS), brexanolone (allopregnanolone, AlloP), ganaxolone and zuranolone, have been approved for the treatment of neuropsychiatric illnesses including postpartum depression and seizures in a neurodevelopmental syndrome. The approved agents are pregnane steroids and strong positive allosteric modulators (PAMs) of gamma-aminobutyric acid type A receptors (GABA_A_Rs). Broad effects on GABA_A_Rs play important roles in therapeutic benefits. However, these NAS also have actions on non-GABAR targets that could be important for clinical outcomes. Thus, understanding the broader effects of NAS is potentially important for expanding the therapeutic landscape of these important modulators. The approved NAS as well as other structurally distinct NAS and oxysterols have effects on non-GABA_A_R receptors and ion channels, along with intracellular actions that could have therapeutic importance, including modulation of cellular stress mechanisms, neuroinflammation, mitochondrial function and autophagy, among others. In this review, we explore GABAergic and other cellular effects of pregnane steroids including novel molecules that have potential therapeutic importance. This work discusses the complex chemical nature of NAS and what is being learned at cellular, molecular, synaptic and brain network levels about key sites of action including GABA_A_Rs and other targets.

## 1. Introduction

Psychiatric illnesses cause substantial disability and death across the human life span with major depressive disorder (MDD) and anxiety disorders being leading causes of disability worldwide (Vigo et al., 2016; Caine, 2017). The COVID-19 pandemic increased recognition of depression, anxiety, and related disorders as major public health concerns (Mahmud et al., 2023). While current antidepressants are beneficial, these treatments have significant side effects and are ineffective in up to a third of MDD patients, with relapse to illness complicating the course of many who respond initially (Conway et al., 2017; Rush et al., 2022). Thus, development of novel and more effective antidepressant treatments is a huge unmet need. The success of the racemic dissociative anesthetic ketamine and one of its enantiomers, esketamine, and the advent of neuroactive steroids (NAS) as rapidly acting antidepressants gives hope for identifying additional treatments with novel mechanisms of action (Abdallah et al., 2015, 2018; Riggs and Gould, 2021).

The FDA approval of the NAS, brexanolone, an intravenous formulation of the endogenous neurosteroid, 3α-hydroxy-5α-pregnan-20-one (allopregnanolone, AlloP), and the synthetic steroid, zuranolone, to treat postpartum depression (PPD) has spurred new ideas about antidepressant development (Meltzer-Brody et al., 2018; Gunduz-Bruce et al., 2019). A third NAS, ganaxolone, was approved by FDA in 2022 for treatment of seizures in cyclin-dependent kinase-like 5 deficiency disorder, a neurodevelopmental illness. The positive clinical results make it important to understand how NAS produce therapeutic benefits, with the hope of developing more specific and effective psychotropic agents (Maguire, 2019). The duration of therapeutic effects of NAS in PPD (lasting a month or longer after the drug is stopped) (Meltzer-Brody, 2018; Gunduz-Bruce et al., 2019) strongly suggests that the well-established acute allosteric modulation of GABA_A_ receptors (GABA_A_Rs) may not be the sole mechanism of action and indicate the importance of additional, underappreciated mechanisms that impact neural circuits and behaviors. While GABA_A_Rs are major contributors to psychoactive effects of NAS (Maguire, 2019), other GABAergic agents are not typically antidepressant (Gunduz-Bruce et al., 2019). Ion channels other than GABA_A_R chloride channels, notably low-voltage activated (LVA) calcium channels and N-methyl-D-aspartate glutamate receptors (NMDARs), are also engaged by some NAS (Zorumski et al., 2013; 2019; Joksimovic et al., 2018), suggesting their potential importance as therapeutic targets. In addition, intracellular targets related to cellular stress and inflammation may also be important (Rupprecht et al., 1993; Langmade et al., 2006; Jiang et al., 2016; Ishikawa et al., 2021; Balan et al., 2019; Noorbakhsh et al., 2014). A recent broad screen of targets revealed additional channels, transporters, and signaling molecules that could be differentially targeted by NAS of varied structures (Kumar et al., 2025).

**Fig. 1.**
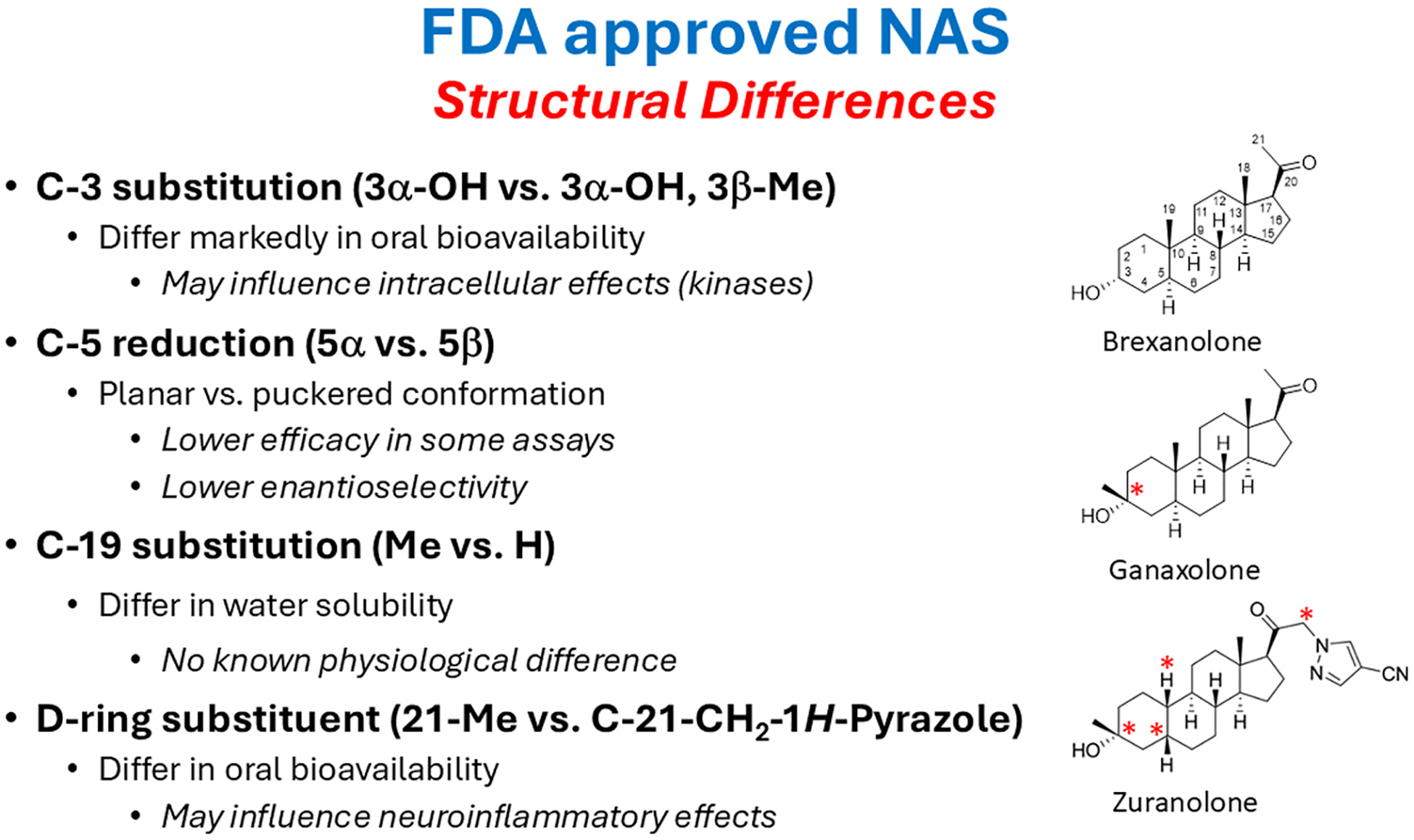
The figure depicts structures of the three NAS that have been FDA approved for clinical use and highlight structural differences among them. Possible effects of the structural differences are also highlighted.

Fig. 1 shows the structure of the three US Food & Drug Administration (FDA) approved NAS and highlights structural differences that will be discussed in this review. We also highlight novel synthetic NAS with unique properties that have potential for development as therapeutic agents, focusing on agents that build on the clinical successes of the approved pregnane steroids. Other tool compounds that are helping to elucidate sites and targets for NAS are also discussed. The terms “neurosteroid” and “NAS” can cause confusion. In this review, we aimed to use “neurosteroid” in its original meaning as an endogenous molecule made in the nervous system (Corpechot et al., 1981) and “NAS” as a broader category of endogenous and synthetic steroids that modulate nervous system function (Paul and Purdy, 1992). However, even this usage is vague in that AlloP is a prototypical neurosteroid but when formulated as brexanolone is technically a NAS. Table 1 lists exemplar neurosteroids and oxysterols discussed in this review and NAS derived from those agents that have proceeded to human clinical trials. Most compounds described in this paper are NAS, and for purposes of stylistic flow, we sometimes use NAS as an inclusive, umbrella term.

## 2. Literature search

We conducted literature searches using PubMed and Chemical Abstracts with search terms that included psychiatry, neurosteroids, neurosteroid enantiomers, neuroactive steroids, allopregnanolone, pregnanolone, androgens, and sulfated steroids. We refined the search returns to emphasize work done with pregnane steroids (progestogens) in psychiatry. We also draw upon the collective expertise of the authors who have more than 30 years of experience studying the chemistry and biology of neurosteroids.

## 3. Overview of NAS as novel therapeutics

Neurosteroids endogenously and powerfully modulate major targets in brain, affecting ligand-gated and voltage-gated ion channels that regulate neuronal excitability, intercellular communication and plasticity. There is growing evidence that NAS also influence cellular responses to stress and inflammation. Thus, NAS reflect the increasing appreciation that highly effective therapies are likely to involve multiple targets. The broad actions of NAS suggest that they may benefit a range of neuropsychiatric illnesses, including major depression, anxiety disorders and other stress-related disorders (Maguire and Mennerick, 2024; Schule et al., 2014; Zorumski et al., 2013; 2019). In addition to representing attractive therapeutic leads, neurosteroids are synthesized locally in brain from cholesterol, particularly within excitatory neurons, and their synthesis is altered by acute cellular and environmental stressors (Purdy et al., 1991; Tokuda et al., 2011; Ishikawa et al., 2014). A major class of these steroids includes AlloP, a progesterone metabolite (Paul and Purdy, 1992). AlloP, formulated as brexanolone in a β-cyclodextrin (Captisol) for intravenous infusion, was approved for clinical use in PPD by the FDA in 2019 (Meltzer-Brody et al., 2018; Meltzer-Brody and Kanes, 2020). A related, but structurally distinct synthetic, orally active NAS, zuranolone (Deligiannidis et al., 2021), was approved for PPD by FDA in 2023 and has had successful pivotal trials for MDD (Gunduz-Bruce et al., 2019). Importantly, brexanolone and zuranolone are GABA_A_R positive allosteric modulators (PAMs) and have faster onset of action and possibly longer duration of response compared to current antidepressants even when administered in pulses over relatively short periods. It remains unclear, however, whether brexanolone and zuranolone represent the optimal NAS-like drugs. For instance, sedation is a potentially problematic side effect (Meltzer-Brody et al., 2018) and FDA denied approval of zuranolone for major depression based on clinical trials to date. To enhance the trajectory of NAS clinical development, a better understanding of molecular, cellular, neuro-circuit, and behavioral effects is needed.

AlloP is a potent, effective, and broad-spectrum GABA_A_R PAM that enhances both tonic (extrasynaptic) and phasic (synaptic) GABAergic inhibition (Belelli et al., 2020; Mogdil et al., 2017; Lu et al., 2020; Shu et al., 2012). These effects contribute significantly to psychotropic actions (Maguire and Mennerick, 2024). At low concentrations, AlloP and NAS of similar structure may have selective actions at GABA_A_Rs expressing δ subunits that are primarily expressed extrasynaptically (Herd et al., 2007), although some studies have emphasized the broad-spectrum effects of NAS at most if not all GABA_A_R subtypes (Shu et al., 2012; Lu et al., 2020).

NAS, acting at δ-containing GABA_A_Rs, modulate local oscillations in basolateral amygdala and affective-like behaviors in the stressed rodent (Antonoudiou et al., 2022; Walton et al., 2023). Nevertheless, δ-containing GABA_A_Rs may not fully explain antidepressant actions, so it is important to consider how GABAergic effects in combination with other potential mechanisms could contribute to therapeutic benefits. For instance, NAS relieve stress and depressive-like behaviors in female δ-deficient mice (Melon et al., 2018; Antonoudiou et al., 2022). Thus, GABA_A_Rs may not be the only relevant cellular target of AlloP, and some steroids have primary actions that engage alternative targets. For example, certain sulfated NAS are negative modulators of NMDARs (Park-Chung et al., 1997) and thus could have ketamine-like effects. Unfortunately, most sulfated steroids exhibit low NMDAR potency and are also inhibitors of GABA_A_Rs. Synthetic efforts have resulted in tool compounds that combine potent NMDAR negative allosteric actions and AlloP-like GABA_A_ positive allosteric actions (Mennerick et al., 2001). These compounds are unique and may represent agents with clinically fortuitous dual mechanisms of action. It remains unclear, however, whether pharmacokinetic properties of these compounds will permit therapeutic development.

Other work led to the observation that LVA (T-type) calcium channels may be important targets for certain neurosteroids and NAS, including AlloP, acting as LVA inhibitors (Todorovic et al., 2004; Atluri et al., 2018). Synthetic chemistry efforts have generated NAS with actions at T-channels but not GABA_A_Rs (Pathirathna et al., 2005a), while other compounds have selective actions at GABA_A_Rs but not LVA channels. NAS modulation of LVAs in subiculum, the major output path of the hippocampus, shapes the activity and plasticity of these neurons (Joksimovic et al., 2019). However, it remains unclear how different constellations of actions at LVA and GABA_A_Rs affect inter-regional connectivity and animal behavior.

**Fig. 2.**
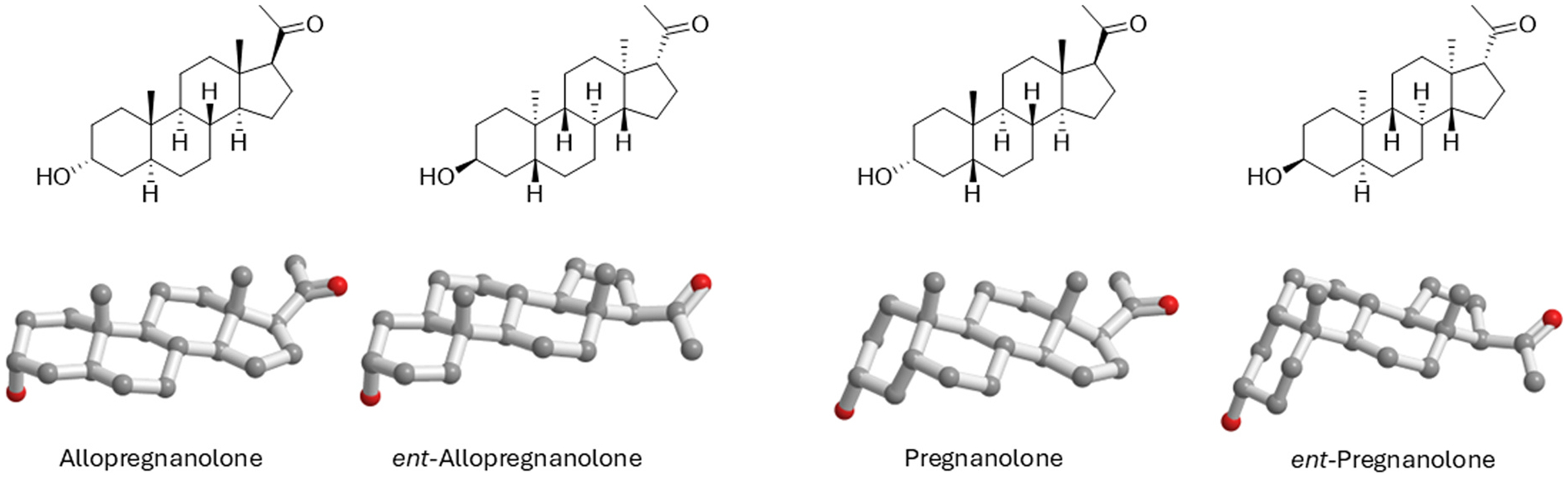
2D and 3D Structures of natural allopregnanolone, pregnanolone and their unnatural enantiomers. 2D structures are in the top row with 3D structures in the bottom row. The mirror plane in 2D structures is above the page with the viewer looking up from the natural steroids. For display purposes, the 2D enantiomer pairs are displayed in the plane of the page. In 2D it is difficult to ascertain that the 3β-hydroxyl group of the unnatural enantiomer is still in the axial configuration as found in its natural enantiomer, not in the equatorial configuration as it would be in a natural 3β-hydroxysteroid. This becomes clear when the enantiomer pairs are shown as 3D structures. 3D structures in the bottom row have the mirror plane as the plane of the page with the viewer looking along a steroid edge wherein the natural structures would be in front of the plane of the page and unnatural structures behind the plane of the page. As for the 2D structures, the 3D enantiomers pairs are displayed in the plane of the page. A superimposition of the 3D enantiomer pairs would overlay the 3-axial hydroxy groups, the steroid A and C rings as well as the 18 and 19 methyl groups, but not the steroid B and C rings.

Fluorescent NAS and photoaffinity labels (PALs) demonstrate that NAS accumulate readily within cells, interacting, in the case of AlloP derivatives, with Golgi, mitochondria, microtubules, and other intracellular targets (Akkerman et al., 2024; Chen et al., 2012a; Jiang et al., 2016). Because GABAergic agents currently in clinical use (e.g. benzodiazepines, barbiturates, propofol) are not known to be effective antidepressants, we hypothesize that these intracellular targets may contribute directly or indirectly to psychotropic effects. Current evidence suggests that neurosteroid and NAS targets may include proteins important for cellular stress responses, including autophagy and neuroinflammation (Kim et al., 2012; Ishikawa et al., 2021; Balan et al., 2019; 2021). Unique NAS, most notably unnatural enantiomers of pregnane steroids (Fig. 2), also engage intracellular signaling pathways without substantially affecting ion channel targets (Wittmer et al., 1996; Hu et al., 1997; VanLandingham et al., 2006; Covey et al., 2000; 2023). At the molecular level, PALs have facilitated the identification of several proteins that represent candidates to mediate intracellular effects (discussed below). To understand the impact of neurosteroids and NAS with different combinations of actions, we will need to understand how the constellation of actions conspire to affect local and inter-regional brain function and relevant behaviors.

## 4. Next gen NAS with therapeutic potential

### 4.1. GABAAR Negative Allosteric Modulators (NAMs)

**Fig. 3.**
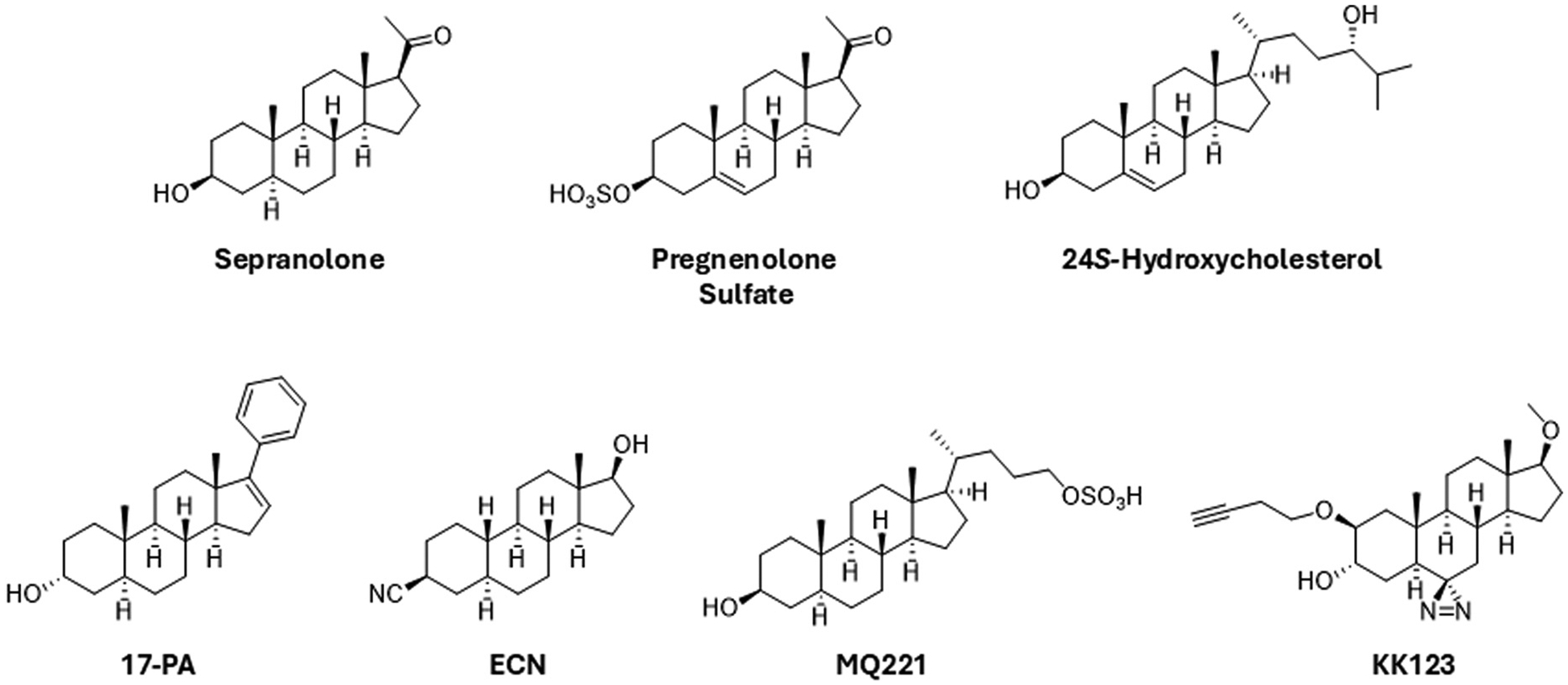
The figure displays 2D structures of exemplar steroids described in the text.

A recurring theme in the biology of neuropsychiatric illnesses is that these disorders involve changes in the balance of excitation (E) and inhibition (I) in local brain regions and neural circuits (Luscher et al., 2011; Luscher and Mohler, 2019). As a broad category, NAS are ideally positioned to play key roles in modulating E/I balance both endogenously and exogenously, making them potentially viable therapeutic targets. Most interest and success to date has focused on the class of NAS that are PAMs of GABA_A_Rs. As noted, the prototype for these agents is AlloP (brexanolone), but GABA PAM activity is shared by other 5α-reduced (planar) neurosteroids and by certain 5β-reduced (puckered) NAS (e.g. pregnanolone and zuranolone) (Fig. 2). Importantly, GABA PAMs typically have a hydrogen bond donor in the 3α-position and a hydrogen bond acceptor in the 17β-position (Zorumski et al., 2013, 2019). A different class of neurosteroid/NAS acts as NAMs at GABA_A_Rs and exhibit activation-dependent, non-competitive inhibition. Examples include certain steroids that are sulfated at the C3-position of the steroid A-ring and include pregnenolone sulfate (PREGS) and dehydroepiandrosterone sulfate (DHEAS) (Eisenman et al., 2003; Seljeset et al., 2018; Shen et al., 2000; Pierce et al., 2021) (Fig. 3). Sulfated neurosteroids/NAS can also modulate NMDARs with some serving as PAMs and others as NAMs (discussed below). Steroids that have a 3β-hydroxyl group are also GABA_A_R NAMS at low micromolar concentrations (Wang et al., 2002). Interestingly, 3α-hydroxy steroids that are typically PAMs can also inhibit GABARs at higher concentrations and under conditions of receptor saturation (Pierce et al., 2024).

Neurosteroid/NAS GABA_A_R NAMs are activation-dependent, meaning that they have little effect at low GABA concentrations but are effective antagonists at higher GABA levels (as at synapses) or in the presence of GABA PAMs (including AlloP). Because 3β-hydroxy NAS antagonize effects of AlloP without significant effects on lower-level GABAergic activity, they are sometimes referred to as “GABA_A_R modulating steroid antagonists” (GAMSAs) (Johansson et al., 2016, 2018). This designation is not strictly accurate because these NAS can also dampen the effects of other GABA_A_R PAMs (Wang et al., 2002). There are, however, certain synthetic NAS that have little or no intrinsic activity on their own but inhibit the actions of NAS PAMs without altering other non-steroidal GABA PAMs. An example of such a “neutral allosteric ligand” (NAL) is (3α,5α)-17-phenyladrost-16-en-3-ol (17-PA) (Mennerick et al., 2004) (Fig. 3). Interestingly, 17-PA antagonizes 5α-reduced PAMs but not 5β-reduced PAMs, potentially reflecting differences in how 5α- and 5β-reduced PAMs engage GABA receptors.

Consistent with the activation-dependence of the GABA NAMs, these agents can enhance GABA_A_R desensitization (Shen et al., 2000; Eisenman et al., 2003; but see Pierce et al., 2021). Their ability to dampen GABA activity can result in disinhibition and increase the E/I ratio. Disinhibition has been described with certain rapidly acting antidepressants, including ketamine and nitrous oxide (Widman and McMahon,2018; Zorumski et al., 2015; 2016), suggesting that GABA NAMs could have mood altering properties. Reducing GABA_A_R-mediated inhibition increases transcription of BDNF, a neurotrophic factor linked to antidepressant-like effects of ketamine in preclinical models (Horvath et al., 2021). Furthermore, a non-steroidal GABA NAM acting at a benzodiazepine site on α5 subunit expressing GABA_A_Rs in the CA1 hippocampal region has anti-anhedonic effects in a rodent stress model and restores stress-induced decreases in excitatory synaptic strength in temporoammonic inputs to CA1 (Zanos et al., 2017; Troppoli et al., 2022; Thompson, 2024). The α5 NAMs also appear to have cognitive enhancing effects, which could be beneficial in psychiatric illnesses. These results provide important proof-of-concept that GABA NAMs could be potential antidepressants.

Clinical interest in 3β-hydroxysteroid GABA NAMs has centered on disorders in which there is high GABA activity and/or high levels of endogenous AlloP, including liver diseases such as hepatic encephalopathy and primary biliary cholangitis. The steroid golexanolone (GR3027, 3α-ethynyl-3β-hydroxyandrostan-17E-one oxide) is under investigation for these latter indications (Montagnese et al., 2021; Backstrom et al., 2024). A psychiatric syndrome of interest is premenstrual dysphoric disorder (PMDD), a complex syndrome that may involve altered sensitivity to AlloP (Hantsoo and Epperson, 2020). Clinical evidence suggests that PMDD may respond to treatment with agents that inhibit 5α-reductase (5AR), a key enzyme in synthesis of AlloP (and other endogenous steroids) (Martinez et al., 2016). There is also interest in the potential clinical utility of 5AR inhibitors in other disorders involving stress, impulsivity, dopamine dysregulation or elevated levels of AlloP, including opiate use disorder (Bosse et al., 2021), tic disorders (Branca and Bortolato, 2024; Caddedu et al., 2023) and other neuropsychiatric illnesses (Scheggi et al., 2024).

In part because of the possible role of AlloP in PMDD, a 3β-hydroxy NAS is under investigation as a potential treatment. The steroid of most interest to date has been sepranolone (UC1010, 3β-hydroxy-5α-pregnan-20-one), also called isoallopregnanolone, the 3β-hydroxy epimer of AlloP (Bixo et al., 2017; 2018) (Fig. 3). There may also be a therapeutic use for AlloP or other PAMs in PMDD and in mood disorders in early pregnancy even though AlloP levels may be elevated (Hantsoo and Epperson, 2020; Wenzel et al., 2021). In these latter conditions, there is also interest in whether NAS GABA NAMs have antidepressant effects. To date, the 3β-hydroxy NAMs appear to be well tolerated but clinical outcome results have been mixed (Backstrom et al., 2021a,b).

### 4.2. T-type calcium channel antagonists

AlloP and select NAS modulate ion channels other than GABA_A_Rs and NMDARs. Certain NAS inhibit low voltage activated (LVA) calcium channels (T-type channels). Because of their unique activation voltages and transient (deactivating) currents, T-channels are important for burst firing in certain brain regions and help regulate oscillatory firing that contributes to rhythmic neural activity. AlloP is a partial inhibitor of T-channels at concentrations that overlap with those that potentiate GABA_A_Rs, with an IC_50_ of 0.9 μM for LVA channels in rodent dorsal root ganglion neurons. Effects on T-channels, like effects on GABA_A_Rs, are enantioselective with the unnatural AlloP enantiomer being at least an order of magnitude less potent than the natural neurosteroid (Pathirathna et al., 2005a,b). As noted, T-channels contribute to burst firing, and increases in bursting in lateral habenula neurons, a hub in networks underlying negative emotion (Cui et al., 2019), contributes to depressive-like behaviors in rodents. Inhibiting T-channels, including directly in lateral habenula, has antidepressant-like effects and may contribute to the actions of ketamine (Yang et al., 2018).

AlloP and a NAS with more selective effects on T-channels ((3β,5β,17β)-3-hydroxyandrostane-17-carbonitrile; also called 3β5βACN, 3β-OH or B260 in other publications) (Todorovic et al., 2004; Tat et al., 2020) inhibit burst firing in pyramidal neurons in the subiculum (Joksimovic et al., 2019). LVA channels also contribute to long-term potentiation (LTP) in the subiculum but not in nearby hippocampal area CA1. Effects on T-channels may also contribute to sedation induced by B260 and the endogenous neurosteroid, epipregnanolone (3β-hydroxy-5β-pregnan-20-one), with effects on the Cav3.1 subtype playing an important role (Coulter et al., 2021; Stamenic et al., 2021). These observations indicate that effects on T-channels and burst firing could be important for certain clinical effects of NAS, including sleep and antidepressant actions. It is also notable that B260 and epipregnanolone are 3β-hydroxy steroids and share inhibitory effects on GABA_A_Rs noted above at higher concentrations. Interestingly, B260 is metabolized endogenously to a 3α-hydroxy steroid with GABAergic effects that contribute to hypnotic actions; this conversion occurs faster in female rodents (Manzella et al., 2023).

Structure-activity studies have revealed other unique NAS that help disentangle effects of T-channels from GABA_A_Rs. (3β,5α,17β)-17-hydroxyestrane-3-carbonitrile (ECN) is a partial T-channel inhibitor, which, unlike AlloP, does not potentiate synaptic GABA_A_Rs (Fig. 3). This agent enantioselectively inhibits LVA currents with an IC_50_ below 1 μM (Todorovic et al., 1998). Another novel steroid (CDNC24) potentiates GABA receptors but has no effect on T-channels. In rodents, ECN, but not CDNC24, is analgesic in non-pathological conditions (Pathirathna et al., 2005a,b), but both compounds show analgesia in a sciatic nerve ligation model and in experimental diabetic neuropathy (Pathirathna et al., 2005b; Latham et al., 2009). This suggests that both T-channels and GABA_A_Rs can contribute to analgesia. Other studies indicate that T-channel inhibitors with or without GABA effects may be useful for pre-emptive treatment of surgical pain. Taken together, effects on T-channels may be important to consider in potential psychiatric indications (Joksimovic et al., 2018; Manzella et al., 2022).

### 4.3. NMDAR NAMs & agents with dual effects on GABA and NMDA receptors

The success of ketamine in major depression, particularly treatment resistant depression, has piqued interest in whether NAS NMDAR NAMs could be developed as antidepressants and possibly neuroprotectants. In addition, NMDAR PAMs have potential for development as cognitive enhancers and/or treatments for disorders associated with NMDAR hypofunction including schizophrenia and anti-NMDAR encephalitis, along with certain neurodevelopmental syndromes (Kudova, 2021).

Neurosteroids/NAS can act as either PAMs or NAMs at NMDARs depending on their structures and the subunit composition of the receptors. For most NMDAR-active NAS, the presence of a charged group at the C3 position helps distinguish these steroids from GABA_A_R PAMs, although sulfated steroids have NAM actions at GABA_A_Rs. Differences in effects on NMDARs are highlighted by two endogenous neurosteroids, pregnanolone sulfate (PAS), an NMDAR NAM, and pregnenolone sulfate (PREGS), an NMDAR PAM (Wu et al., 1991). Both steroids feature a C3 sulfate in the 3α conformation for PAS and 3β-conformation for PREGS (Fig. 3). However, the conformation at C3 seems less critical in determining the type of response compared to the conformation at C5. 5β-Reduction at C5 as in PAS results in a puckered (bent) conformation and NMDAR NAM activity (Fig. 2). In contrast, a flat (planar) structure results typically in PAM activity as observed with 5α-reduced steroids or pregn-5-enes akin to PREGS (Park-Chung et al., 1994; 1997; Weaver et al., 2000). The situation is also complicated by differing effects of sulfated NAS depending on NMDAR subunit composition (Malayev et al., 2002). Another challenge in developing sulfated NMDAR-modulating steroids as therapeutics is that these agents have relatively low potency compared to GABAergic NAS and more restricted blood-brain barrier permeability. The physiological significance of the endogenous sulfated neurosteroids, especially at NMDARs, also remains uncertain because of discrepancies in reported brain levels (Gibbs et al., 2006), but this does not preclude potentially therapeutic pharmacological actions. Specific sites of action of sulfates on NMDARs are not certain although PREGS appears to interact with the receptor ligand-binding domain and transmembrane regions (Horak et al., 2006; Wilding et al., 2016).

The success of ketamine and NAS as antidepressants with unique and different mechanisms raises questions about whether agents with dual NMDAR NAM and GABA PAM actions would be clinically advantageous. The NAS chemical platform offers opportunities to explore this possibility, and we have identified two such steroids to date. The first was 3α,5β−20-oxo-pregnane-3-carboxylic acid (3α5β-PC) (Mennerick et al., 2001). This steroid inhibits NMDARs and potentiates GABA_A_Rs at concentrations below 10 μM and at negative membrane potentials. However, 3α5β-PC also inhibits GABA_A_Rs at higher concentrations, with depolarization and high extracellular pH increasing the block. The pH-dependence of block suggests that the un-ionized molecule likely is important for GABA potentiation, consistent with other structure-activity studies (Covey et al., 2001; Akk et al., 2007). More recently, another steroid with dual GABA_A_R and NMDAR effects has been described. This steroid (called MQ-221) is a 3β-hydroxy, 5α-reduced molecule that features a sulfate attached to a side chain off the D-ring (the opposite end to typical sulfated NAS) (Fig. 3). MQ-221 inhibits NMDARs while potentiating GABA_A_R currents under physiological conditions (Ziolkowski et al., 2021). GABA potentiation by this compound is surprising, given its 3β-hydroxy structure. MQ-221, however, also exhibits activation-dependent GABA_A_R inhibition at higher concentrations, akin to other 3β-hydroxy and sulfated steroids described above, making its overall effects complex. Behavioral effects of these two unique NAS have not yet been determined.

### 4.4. Oxysterols & NMDARs

Certain side chain oxidized derivatives of cholesterol are also important modulators of ion channels and brain function in particular NMDARs. 24S-Hydroxycholesterol (24S-HC) is a potent and effective endogenous NMDAR PAM (Paul et al., 2013; Linsenbardt et al., 2014; Sun et al., 2016a) that interacts with membrane spanning regions of NMDARs (Wilding et al., 2016) and promotes synaptic plasticity (Fig. 3). A synthetic analogue of 24S-HC can overcome the impact of cellular stressors on hippocampal plasticity and learning (Izumi et al., 2021b) and can dampen the effects of psychotomimetic drugs in rodents (Paul et al., 2013). These oxysterols also prevent and correct NMDAR receptor changes associated with patient-derived anti-NMDAR autoantibodies in preclinical models (Warikoo et al., 2018; Mannara et al., 2020).

The enzyme that synthesizes 24S-HC, CYP46A1, is brain specific and largely expressed in excitatory neurons (Russell et al., 2009). There is considerable interest in whether changes in levels of 24S-HC and CYP46A1 contribute to neuropsychiatric illnesses, particularly neurodegenerative disorders such as Alzheimer’s disease, Huntington’s disease and others (Sun et al., 2016b). Intriguingly, CYP46A1 activation stimulates TrkB receptors (Sodero et al., 2011a; Moutinho et al., 2016), a target of BDNF and a possible mechanism of certain antidepressants (Krystal et al., 2024). Effects of CYP46A1 on TrkB receptors are independent of BDNF (Sodero et al., 2011a). It is presently uncertain whether TrkB activation results from effects of 24S-HC or associated decreases in cellular and membrane cholesterol. Efavirenz, a clinically-used antiviral agent, stimulates CYP46A1 and increases 24S-HC levels (Pikuleva and Cartier, 2021).

Another oxysterol, 25-hydroxycholesterol (25HC), is an important inflammatory modulator in the periphery where its synthetic enzyme, cholesterol-25-hydroxylase (Ch25H), is largely expressed in monocytes. Ch25H is also expressed in brain, particularly in microglia (Zhang et al., 2014), and brain levels of the enzyme and 25HC increase dramatically during neuroinflammatory stimulation (Wong et al., 2020; Izumi et al., 2021a). 25HC is a weak partial agonist at a putative oxysterol site on NMDARs and functionally antagonizes more effective PAMs such as 24S-HC (Linsenbardt et al., 2014). Recent evidence indicates that activation of Ch25H and increases in 25HC contribute significantly to effects of pro-inflammatory stimulation on hippocampal plasticity and learning (Izumi et al., 2021; 2024).

Given the importance of changes in cognitive function in a wide range of neuropsychiatric disorders and increasing evidence that inflammation contributes to the pathogenesis and outcomes of these illnesses, there is interest in whether synthetic derivatives of 24S-HC and 25HC and/or modulators of their synthetic enzymes can be developed for therapeutic purposes (Geoffroy et al., 2022). Indeed, a synthetic analogue of 24S-HC, dalzanemdor, (Beckley et al., 2024), made it into in human clinical trials as a potential cognitive enhancer (Gray et al., 2024; Koenig et al., 2024), but recently failed three Phase 2 trials for cognition in neurodegenerative illnesses. Inhibitors of CYP46A1 such as soticlestat are also in development as possible treatments for epilepsy (Nishi et al., 2020; Pikuleva and Cartier, 2021; Popiolek et al., 2020).

It is also important to consider possible interactions of oxysterols with the endogenous pregnane neurosteroids that are the primary focus of this review. In stressed cells, accessible cholesterol is mobilized to promote homeostasis (Das et al., 2014; Sodero et al., 2011a,b) and converted to 25HC or 24S-HC in a single enzymatic step depending on cell type. These oxysterols promote further cholesterol mobilization (Heisler et al., 2023) and trafficking from endoplasmic reticulum to mitochondria where cholesterol undergoes side chain cleavage to form pregnenolone (Barbero-Camps et al., 2014), the first and rate-limiting step in synthesis of neurosteroids including AlloP (Ishikawa et al., 2018; Mast et al., 2020). A variety of stressors promote cholesterol mobilization and AlloP synthesis, but a common factor in neurons involves activation of NMDARs (Brachet et al., 2015; Sodero et al., 2011a, b; Ishikawa et al., 2014; 2018; Tokuda et al., 2011; Zorumski and Izumi, 2012). AlloP in turn serves as a homeostatic regulator of oxidative stress (Lejri et al., 2017), neuronal death (Ishikawa et al., 2014), mitochondrial function (Grimm et al., 2014), autophagy (Ishikawa et al., 2021, 2022) and neuroinflammation (Balan et al., 2019; 2021; Izumi et al., 2024).

### 4.5. NAS enantiomers

The effects of AlloP and other 3α5α GABA_A_R PAMs are highly enantioselective with the natural enantiomers showing 10-fold or greater sensitivity for potentiating or directly activating GABA channels (Wittmer et al., 1996; Covey et al., 2001; 2023) (Fig. 2). Similar high enantioselectivity has been observed for anesthetic effects in animals. 5β-Reduced PAMs also show enantioselectivity but separation between the enantiomers is lower (about 3-fold separation in the case of pregnanolone, the 5β-reduced analogue of AlloP) (Covey et al., 2000) (Fig. 2). Enantiomeric steroids are important because they have identical physical-chemical properties and differ only in their rotation of polarized light (Alakoskela et al., 2007). This stands in contrast to diastereomers (e.g. 3α vs. 3β NAS), which have different physical-chemical properties. Importantly, enantiomers are useful in determining the role of chiral environments in effects of NAS. Enantiomeric pairs exhibit marked differences on GABA_A_Rs but similar effects on cell membranes and intracellular accumulation (Chisari et al., 2009, 2010; Jiang et al., 2016; Covey et al., 2023).

Similar to GABA_A_R PAMs, the ability of certain NAS to inhibit LVA calcium channels also shows high enantioselectivity with the natural steroid absolute configuration exhibiting greater potency. However, enhanced potency of natural NAS enantiomers is only relative, and structure-activity studies have demonstrated that certain unnatural enantiomers have higher activity at GABA_A_Rs than their natural counterparts (Katona et al., 2008; Krishnan et al., 2012; Qian et al., 2014). Examples include the enantiomers of etiocholanolone, where natural etiocholanolone has a minor effect on GABA_A_Rs while the unnatural enantiomer is highly active. These observations are important because they open a potential avenue for novel drug development (Covey et al., 2023). Because unnatural enantiomers are likely metabolized differently from natural steroids, it may be possible to design active molecules at GABA_A_Rs or other targets that have longer durations of action compared to endogenous steroids and lower likelihood of biotransformation to related active endogenous steroids. Indeed, active unnatural enantiomers of androsterone and etiocholanolone exhibit longer durations of anticonvulsant activity in rodents (Zolkowska et al., 2014).

Beyond effects on ion channels, certain other actions of NAS show little enantioselectivity and some non-enantioselective effects could be important in neurotherapeutics. For example, neuroprotective effects show little enantioselectivity in models of Niemann-Pick type C (NPC) disease (Langmade et al., 2006) and traumatic brain injury (TBI) (VanLandingham et al., 2006). Furthermore, anti-inflammatory effects of NAS may be important in their therapeutic effects in multiple neuropsychiatric illnesses, including depression, and there is evidence that effects on microglia and pro-inflammatory changes of AlloP are not enantioselective (Langmade et al., 2006; Izumi et al., 2024). Perhaps related to these effects and to neuroprotective effects of NAS, it also appears that activation of pregnane X receptors (PXR), which can regulate microglial activation and production of BDNF, is not enantioselective (Langmade et al., 2006). AlloP is highly protective in *ex vivo* and *in vivo* glaucoma models and this effect is also not enantioselective (Ishikawa et al., 2021). Interestingly, AlloP enantiomers appear to work by overlapping but distinct mechanisms in the retina (Izumi et al., 2023). Both activate macroautophagy and this contributes to retinal protection, but only the natural AlloP enantiomer is protective via GABA_A_Rs (Ishikawa et al., 2021; 2022).

## 5. Alternative & intracellular effects of neurosteroids/NAS

In addition to direct effects on plasma membrane receptors and channels, AlloP and other GABAergic NAS promote expression and trafficking of GABA_A_R subunits with prominent effects on extrasynaptic receptors and tonic inhibition in dentate gyrus granule cells. Effects on receptor trafficking/insertion involve phosphorylation of specific subunits by protein kinase C (PKC) and other kinases (Abramian et al., 2014), although mechanisms underlying kinase activation and receptor trafficking are not certain. Not all NAS enhance tonic inhibition via changes in receptor expression and trafficking. For example, increased receptor surface expression is observed with AlloP but not with the synthetic NAS, ganaxolone, a close structural analogue of AlloP that features a 3β-methyl group to hinder metabolism of the 3α-hydroxy group (Modgil et al., 2017) (Fig. 1). Changes in GABA_A_R expression provide a mechanism by which NAS can have lasting effects on brain function that persist beyond drug exposure and could contribute to psychotropic effects following brief courses of NAS in patients. Membrane progesterone receptors (mPRs) are poorly understood G-protein-coupled receptors expressed in brain that are activated by AlloP and coupled to pathways that could alter protein phosphorylation (Thomas and Pang, 2012). A novel steroid, ORG OD 02–2, appears to be a selective agonist at mPRs without direct effects on GABA_A_Rs. This steroid enhances receptor phosphorylation via cyclic AMP-dependent protein kinase (PKA) and PKC resulting in increased GABA_A_R surface expression and enhanced tonic GABA currents (Parakala et al., 2019).

NAS are highly lipophilic and readily accumulate in cell membranes and at intracellular targets following exogenous administration (Shu et al., 2004; Akk et al., 2005; Jiang et al., 2016; Chen et al., 2018; Chisari et al., 2019). The role of the intracellular pools of NAS is not well understood but represent potential targets that could contribute to therapeutic effects (Li et al., 2007; Jiang et al., 2016). While AlloP and derivatives do not have prominent actions on classical nuclear steroid hormone receptors, metabolism to other steroids (e.g., 5α-dihy-droprogesterone) could result in progesterone-like effects on gene expression (Rupprecht et al., 1993). To date, several intracellular NAS targets have been identified. These include voltage-dependent anion channels 1 and 2 (VDAC-1 & – 2) (Darbandi-Tonkabon et al., 2003; 2004) and β-tubulin (Chen et al., 2012a). VDACs regulate outer mitochondrial membrane permeability and perhaps the mitochondrial permeability transition pore (mPTP) that helps to control movement of molecules from mitochondria to cytoplasm. VDACs also modulate inflammation and cell death pathways. AlloP can inhibit the mPTP and this may contribute to neuroprotective effects (Sayeed et al., 2009). NAS PALs interact with glutamate-73 in VDAC-1 (Darbandi-Tonkabon et al., 2003), but it is presently unknown whether this interaction contributes to changes in mPTP or other effects on cellular function (Cheng et al., 2019); effects on VDAC do not contribute to anesthetic effects or modulation of GABA_A_Rs (Darbandi-Tonkabon et al., 2004). Glu-73 along with threonine-83 and three other sites are also labeled by photoaffinity mimics of cholesterol, suggesting a general regulatory mechanism for steroids on VDAC (Budelier et al., 2017; Cheng et al., 2019), and sites for NAS and cholesterol overlap (Budelier et al., 2019).

Multiple psychiatric illnesses involve altered mitochondrial function and brain energy metabolism (Manji et al., 2012; Vlaikou et al., 2021). NAS can promote cellular energy production via effects on glycolytic and respiration-mediated metabolism, with AlloP having its most prominent effects on respiration (Grimm et al., 2014). Perhaps related to cellular energy metabolism, AlloP also stimulates autophagy to dampen cellular stress (Liao et al., 2009; Kim et al., 2012; Ishikawa et al., 2021; 2022). Autophagy degrades and recycles cellular components to maintain cellular energy and proteostasis in response to cellular stressors (Galluzzi et al., 2016).

NAS also have anti-inflammatory effects that could contribute to therapeutic actions (Langmade et al., 2006; Noorbakhsh et al., 2014; VanLandingham et al., 2006), including effects on physical and cognitive symptoms (Frank et al., 2021). Recent evidence indicates that effects on inflammation may occur via several toll-like receptors (TLRs) including TLR2, TLR4 and TLR7 but not TLR3 (Balan et al., 2019, 2021; Murugan et al., 2019; Izumi et al., 2024); TLRs are pattern recognition receptors that are important in regulating the function of monocytes and microglia. Effects on TLR4 and TLR7 appear to involve interactions of AlloP with the TLR adapter protein, MyD88 (Balan et al., 2021). Effects of AlloP on TLR4 are observed in monocyte-derived macrophages from both females and males while 3α,5α-THDOC and SAGE-516, a structural analogue of zuranolone, inhibited TLR4 only in cells from females, suggesting that substitution at the steroid D-ring may influence sex-specificity (Balan et al., 2022) (Fig. 1). Effects of AlloP on TLR7 were observed only in females. AlloP also promotes endosomal accumulation of TLR4 and production of IL-10, an anti-inflammatory cytokine, in a sex-specific (male only) and MyD88-independent fashion (Balan et al., 2023). Other work indicates that pregnenolone promotes degradation of the TLR2 and TLR4 adaptor protein TIRAP to dampen secretion of inflammatory cytokines (Murugan et al., 2019). Additionally, a 5β-reduced NAS photolabel binds the microtubule protein, β-tubulin, at cysteine-354, a site for colchicine (Chen et al., 2012a). Colchicine has microtubule destabilizing and anti-inflammatory actions, but it is unknown whether NAS interactions with β-tubulin contribute to anti-inflammatory effects. AlloP and pregnanolone can also dampen inflammation via agonism at Takeda G-protein coupled receptor 5 (TGR5), a bile acid receptor expressed in neurons and astrocytes (Keitel et al., 2010; Backstrom et al., 2024). Other studies indicate that a novel NAS, 3β-methoxypregnenolone, binds microtubule-associated protein 2 and promotes microtubule assembly. These effects are thought to contribute to antidepressant-like effects of this steroid in rodents (Bianchi and Baulieu, 2012). This steroid (also known as MAP4343) has advanced to human clinical trials for depression and shows promise as a novel therapeutic (Barbiero et al., 2022; Daftary et al., 2017; ClinicalTrials.gov: NCT03870776).

MAP4343 is a derivative of pregnenolone, the first step in endogenous synthesis of neurosteroids. Pregnenolone synthesis is increased by stressors and drugs of abuse including ethanol and Δ9-tetrahy-drocannbinol (THC). Intriguingly, pregnenolone inhibits certain effects of THC raising the possibility that this agent could be a treatment for substance use disorders and other stress-related conditions (Vallee et al., 2014). Indeed, another pregnenolone analogue, AEF0117 (3β-(4-methoxybenzyloxy)pregn-5-en-20-one) has progressed to human clinical trials as an allosteric modulator of cannabinoid CB1 receptors for cannabis use disorder (Haney et al., 2023; Tomaselli and Vallee, 2019; ClinicalTrials.gov: NCT03325595, NCT03443895, NCT03717272).

AlloP and related NAS increase expression of certain nuclear receptors including pregnane xenobiotic receptors (PXR) and liver xenobiotic receptors (LXR) (Frye et al., 2012; Irwin et al., 2014), and AlloP and other steroids/sterols are PXR/LXR agonists (Fan et al., 2013; Lamba et al., 2004; Langmade et al., 2006). Effects on PXR/LXR could provide mechanisms by which NAS influence synthesis of other intracellular modulators, including steroids, and regulate cellular stress mechanisms (Rong et al., 2013), contributing to anti-inflammatory and neuroprotective actions (Langmade et al., 2006). Furthermore, effects on PXR/LXR can increase BDNF levels (Almeida et al., 2020; Belelli et al., 2021; Wang et al., 2022). Intriguingly, the primary BDNF receptor, tropomyosin receptor kinase B (TrkB), binds certain antidepressants directly and appears to be regulated by cholesterol (Casarotto et al., 2021). Whether AlloP and other NAS interact directly with TrkB is unknown.

## 6. Insights from photoaffinity labeling

**Fig. 4.**
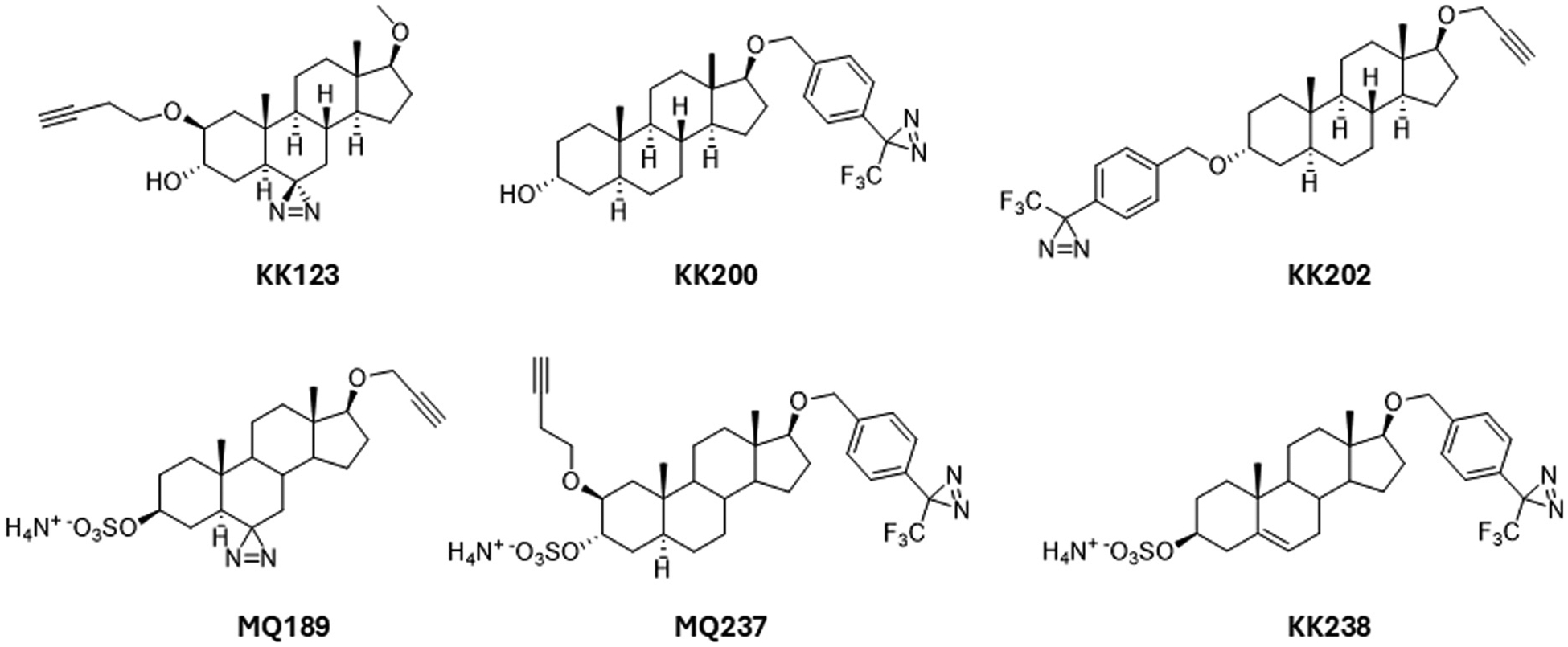
Structures of photoaffinity labeling neurosteroids (PALs). Top row, PALs used in published photoaffinity labeling studies of non-sulfated neurosteroid binding sites on GABARs Chen et al., (2019); Sugasawa et al., (2020)). Bottom row, newly reported PALs that could be used to identify sulfated neurosteroid binding sites on GABARs and NMDARs (Qian et al., 2024).

Efforts to understand the mechanisms by which therapeutic agents interact with specific molecular targets offer great promise for rational drug design. In prior sections of this paper, we described studies that have provided information about sites at which NAS likely act on protein targets. These molecular pharmacology studies benefit greatly from use of PALs, particularly molecules engineered to have structures and actions akin to therapeutic NAS (Chen et al., 2014) (Fig. 4). In this section we discuss recent insights gained from use of PALs.

Studies using site directed mutagenesis provided evidence for direct interactions of NAS PAMs with GABA_A_Rs (Hosie et al., 2006; 2008; Mortensen et al., 2024) at loci involving multiple amino acid residues in transmembrane (TM) regions of heteropentameric receptors. In the first transmembrane region (TM1) of α1 subunits in α1β2γ2 receptors, key loci include glutamine-241 (Q241) and tryptophan-245 (W245), while key sites in TM4 include asparagine-407 (N407) and tyrosine-410 (Y410). These sites contribute to the ability of NAS to enhance the function of GABA_A_Rs. This early work also suggested that direct gating of GABA channels by NAS may involve separate amino acids including threonine-236 (T236) in α1 subunits and tyrosine-284 (Y284) in β2 subunits.

Subsequent studies using PALs to label β3 homopentamers and α1β3 heteropentamers identified phenylalanine-301 (F301) in TM3 of β3 as a NAS target (Chen et al., 2012b; 2019). Other studies using cryo-electron microscopic and x-ray crystallographic structures of chimeric GABA_A_Rs demonstrated that NAS PAMs potentiate and directly gate the receptor via a single interfacial site involving TM3(+) and TM1(−). The NAS 3α-hydroxyl group interacts with Q241 in TM1 along with W245 via hydrophobic stacking of steroid rings. The hydrogen bond acceptor on the steroid D-ring interacts with TM3 at a site analogous to F301 (Laverty et al., 2017). Further, NAS binding sites couple to helices that form the channel pore and modulate a conformation that promotes receptor desensitization (Miller et al., 2017).

Recent studies using novel PALs that allow bio-orthogonal protein labeling by click chemistry provide evidence of even further complexity in NAS interactions with GABA_A_Rs (Fig. 4). In α1β3 pentamers, NAS label at least 7 residues in 3 clusters with intersubunit and intrasubunit interactions (Chen et al., 2019). One cluster of residues localizes near the N407 and Y410 residues in α1-TM4 described by Hosie and colleagues (2006; 2008), consistent with an α1 intrasubunit site between TM1 and TM4. A second cluster in the β3 subunit suggests intrasubunit binding at the extracellular end of the TM3 and TM4 helices, near the Y284 residue identified by Hosie et al. (2006, 2008). A third cluster of labeled residues identified the β3/α1 intersubunit site observed in cryo-electron microscopic studies (Legesse et al., 2023; Sun et al., 2023). Interactions between Q241 in TM1 of α1 and F301 in TM3 of β3 were also identified. Based on mutagenesis experiments, it appears that all of these sites contribute to effects of NAS on GABA_A_Rs with the α1 intrasubunit and β3-α1 intersubunit sites being most critical for PAM activity. These two sites additively and independently contribute to PAM activity, and it does not appear that there are separate sites for receptor potentiation and direct gating.

Studies using other unique PALs provide additional insights into actions on α1β3 receptors. It appears that AlloP, but not its 3β-hydroxy analogue, epi-AlloP, binds the β3(+)-α1(−) intersubunit site that is critical for potentiation. In contrast, both AlloP and epi-AlloP bind the β3 intrasubunit site to promote receptor desensitization, providing insight into activation-dependent inhibition by 3β-hydroxy steroids and complex effects of AlloP. Intriguingly, two other PALs bind all three sites but do not potentiate receptor function; one of these analogues enhances desensitization while the other shows no allosteric modulation (Sugasawa et al., 2020). These PAL analogues provide important information that can help identify more selective ligands at NAS sites, including perhaps design of highly selective agonists and antagonists of steroid actions that have no other allosteric effects (Jayakar et al., 2020). Agents that selectively enhance GABA_A_R desensitization may be particularly important because desensitization has been linked to a form of long-term potentiation of inhibitory synapses, providing a possible underpinning for persisting network effects of NAS (Field et al., 2021).

As noted earlier, the sulfated neurosteroids PREGS and DHEAS are important allosteric modulators that have effects on both GABA_A_Rs and NMDARs. Information about the sites at which these steroids act is limited compared to the GABA PAMs outlined above (Mortensen et al., 2024). Results from cryo-EM studies indicate that PREGS and DHEAS bind within the ion channel pore of GABA_A_Rs where they act as channel blockers (Legesse et al., 2023). Work on identifying the sites of action of these sulfated steroids should be facilitated by the recent synthesis of a series of ten sulfated steroid analogues, five of which have an alkyne group that can be used for click chemistry reactions (Qian et al., 2024). Interestingly, two of these analogies, MQ237 and KK238, are PAMs at GABARs suggesting that these PALs do not bind in the ion channel as this would be expected to inhibit channel activity.

## 7. Beyond sites & cells: effects on brain networks

Current data indicate that psychiatric disorders involve changes in the function of brain networks that underlie emotion, cognition and motivation, with emphasis on alterations in the balance of excitation and inhibition (E/I balance) within and across networks. Because NAS powerfully modulate receptors and ion channels that regulate excitation and inhibition, it is likely that these agents have important effects on brain networks and neural rhythms (Mody and Maguire, 2012; Lambert et al., 2021). There is also considerable evidence that changes in brain oscillations are found in most, if not all major psychiatric illnesses (Buzsaki and Watson, 2012).

Studies of brain network effects of NAS are in their infancy. An early study using magnetic resonance imaging (MRI) in human subjects treated with pregnenolone, a precursor of AlloP and other neurosteroids, indicated that the steroids may alter activity in brain regions related to cognitive control during an emotional appraisal task (Sripada et al., 2013). Changes included dampening of activity in insula and amygdala with enhanced activity in dorsal medial prefrontal cortex (DMPFC) and increased connectivity between amygdala and DMPFC. Presently, there is little information about how NAS affect brain networks in human illnesses. Some evidence indicates that postpartum depression (PPD) is associated with greater connectivity of the DMPFC with other regions of the Default Mode Network (DMN), and that levels of AlloP correlate with intra-DMPFC connectivity (Deligiannidis et al., 2019). Efforts to understand neural network effects of NAS in human illnesses would benefit greatly from use of precision functional neuroimaging methods that provide a framework for mapping brain networks in individual subjects; these tools are rapidly advancing and offer great promise going forward (Gratton et al., 2020; Laumann et al., 2023).

Studies in rodents are providing unique insights into effects on network states potentially relevant to stress and mood. Antonoudiou and colleagues (2022) found that AlloP and synthetic NAS analogues modulate neural oscillations in rats, mice and humans, increasing EEG power over a range of frequencies especially in high theta and beta bands. They further demonstrated a key role for tonic inhibition of parvalbumin-expressing interneurons via δ-subunit expressing (extrasynaptic) GABA_A_Rs in mediating effects of NAS on high theta (6–12 Hz) oscillations in the basolateral amygdala (BLA) of mice. The BLA is important in emotional processing and in regulating emotional behavior; endogenous AlloP in the BLA plays a key role in regulating basal affective tone (Walton et al., 2023; Maguire and Mennerick, 2024). Intriguingly, NAS also protected mice from network and behavioral changes induced by chronic unpredictable stress (CUS), and these effects correlated with changes in BLA theta rhythm. Effects of NAS on network activity and behavior are distinct from the effects of a benzodiazepine, indicating that not all GABA-enhancing agents mimic NAS effects. While results of this study focused on the importance of BLA, CUS is associated with wide-spread changes in brain networks based on functional magnetic resonance imaging studies in mice, and NAS help to restore connectivity across networks (Antonoudiou et al., 2022).

Effects outlined above involve GABA-enhancing NAS. As noted earlier, GABA NAMs may also have psychotropic actions that could lead to novel therapeutic agents. An intriguing aspect of ketamine as a rapid antidepressant is that this agent causes local network disinhibition via effects on interneurons, resulting in persistent enhancement of glutamate transmission in hippocampus and prefrontal cortex (Zorumski et al., 2016). Ketamine increases EEG power in the gamma-frequency range (30–100 Hz), a form of network oscillation driven by interneurons that may contribute to inter-regional brain communication (Lazarewicz et al., 2010; Qi et al., 2018; Lambert et al., 2023). A non-steroidal NAM that acts on GABA_A_Rs expressing α5 subunits in CA1 hippocampal pyramidal neurons that mediate both tonic and phasic inhibition also enhances gamma power and has antidepressant-like effects in rodents (Zanos et al., 2017; Troppoli et al., 2022). Whether NAS GABA NAMs have similar effects is unknown, although gabazine, a GABA_A_R antagonist, induces high theta oscillations in *ex vivo* BLA slices akin to AlloP (Antonoudiou et al., 2022), raising the possibility that NAS PAMs (AlloP) and NAMs may share some mechanisms. In fact, a selective NAM for α1β2δ GABA_A_Rs that mediate tonic inhibition of parvalbumin-expressing interneurons was found to enhance γ-oscillations (Wei et al., 2024).

## 8. Summary & conclusions

The landscape of psychopharmacology is changing dramatically and rapidly. The approval of neurosteroids as therapeutic options offers great hope for identifying novel, more effective and lasting treatments across a range of illnesses. FDA approval of brexanolone and zuranolone for PPD and ganaxolone for developmental seizures are proofs-of-concept. Insights derived from understanding mechanisms of these NAS offer opportunities to identify more powerful treatments going forward. While broad modulation of GABA_A_Rs, including modulation of synaptic receptors that mediate phasic inhibition and extrasynaptic receptors that mediate tonic inhibition, is of major importance in therapeutic effects of GABAR PAMs, it is also important to understand other effects of these agents including actions on other receptors, ion channels and intracellular targets, potentially leveraging NAS with effects that may not include GABAR PAM activity. Whether advances in understanding the molecular pharmacology of NAS on GABA_A_Rs result in highly specific and better treatments remains to be seen. Alternatively, pleiotropic effects including intracellular actions of NAS may prove to be an important part of their broad therapeutic potential and lead to novel treatment approaches.

It is also important to better understand the roles of endogenous neurosteroids as stress-induced modulators in the brain and how changes in their synthesis and actions influence the pathogenesis of symptoms across neuropsychiatric disorders. The endogenous neurosteroids and oxysterols are perhaps best viewed as local homeostatic modulators that help preserve nervous system function, particularly the function of glutamatergic principal neurons, under duress. Other NAS, particularly those that negatively modulate GABA_A_Rs and that negatively and positively modulate NMDARs and voltage-gated ion channels, also offer potential avenues for novel treatments. As highlighted in this review, major progress is being made in understanding the actions of the broad class of pregnane NAS and this work holds great promise for new treatments in psychiatry including treatments based on mechanistic understanding of stress modulation in the brain.

## Figures and Tables

**Table 1:** The table lists endogenous neurosteorids and oxysterols discussed in this review along with NAS derived from those neurosteroids that have been in human clinical trials.

Neurosteroids / Oxysterols	Neuroactive Steroids (NAS)
Allopregnanolone Pregnanolone	Brexanolone Ganaxolone Zuranolone
Pregnenolone	MAP4343 AEF0117
Isopregnanolone Epipregnanolone	Golexanolone (GR3027) Sepranolone (UC1010)
Pregnenolone sulfate Dehydroepiandrosterone sulfate Pregnanolone sulfate	*None in clinical trials*
24S-Hydroxycholesterol	Dalzanemdor (SAGE–718)

## Data Availability

No unpublished primary data were used in this review.

## Abbreviations:

17-PA: 3α,5α−17-phenyladrost-16-en-3-ol
3α5β-PC: 3α,5β−20-oxo-pregnane-3-carboxylic acid
ECN: 3β,5α,17b-17-hydroxyestrane-3-carbonitrile
3β5βACN: 3β-OH or B260, 3β,5β,17β−3-hydroxyandrostane-17-carbonitrile
5AR: 5-alpha reductase
24S-HC: 24S-Hydroxycholesterol
25HC: 25-hydroxycholesterol
AlloP: allopregnanolone
BLA: basolateral amygdala
BDNF: brain derived neurotrophic factor
Ch25H: cholesterol-25-hydroxylase
CUS: chronic unpredictable stress
PKA: cyclic AMP-dependent protein kinase
DMN: default mode network
DHEAS: dehydroepiandrosterone sulfate
DMPFC: dorsal medial prefrontal cortex
E/I balance: excitatory and inhibitory balance
GAMSAs: GABA_A_R modulating steroid antagonists
GABA_A_Rs: gamma-aminobutyric acid type A receptors
LXR: liver xenobiotic receptors
LVA or T-channels: low-voltage activated calcium channels
MDD: major depressive disorder
MPRs: membrane progesterone receptors
MPTP: mitochondrial permeability transition pore
NAMs: negative allosteric modulators
NAS: neuroactive steroids
NAL: neutral allosteric ligand
NMDARs: N-methyl-D-aspartate glutamate receptors
PALs: photoaffinity labels
PAMs: positive allosteric modulators
PPD: postpartum depression
PXR: pregnane X receptors
PAS: pregnanolone sulfate
PREGS: pregnenolone sulfate
PMDD: premenstrual dysphoric disorder
PKC: protein kinase C
TGR5: Takeda G-protein coupled receptor 5
TLRs: toll-like receptors
TM: transmembrane region
TBI: traumatic brain injury
TrkB: tropomyosin receptor kinase B
FDA: US Food & Drug Administration
VDAC-1 & 2: voltage-dependent anion channels 1 and 2
